# Synergistic Effects of HPMC–HEMC Blending on the Rheological Properties of Composite Cement Paste Based on Explainable Machine Learning

**DOI:** 10.3390/ma19143111

**Published:** 2026-07-20

**Authors:** Mingwei Lu, Xin Liu

**Affiliations:** 1 Ningbo Guangtian Jiantong Engineering Management Co., Ltd., Ningbo 315000, China; 2 School of Energy and Power, Jiangsu University of Science and Technology, Zhenjiang 212003, China

**Keywords:** HPMC, HEMC, composite cement paste, rheological properties, explainable machine learning

## Abstract

Fresh composite cement pastes modified with HPMC–HEMC blends were investigated using an explainable machine learning framework to clarify the coupled effects of cellulose ether dosage and blending ratio on rheological performance. A dataset containing 225 mixtures and 11 input variables was established, with fluidity, dynamic yield stress, and plastic viscosity used as target outputs. HPMC and HEMC dosages ranged up to 0.394% and 0.393%, respectively, while the total cellulose ether dosage varied from 0.041% to 0.398%. The results showed that total cellulose ether dosage strongly reduced fluidity but increased yield stress and viscosity, with Pearson correlation coefficients of −0.618, 0.653, and 0.848, respectively. Among the machine learning models, SVR achieved the best fluidity prediction where R^2^ = 0.918, XGBoost performed best for dynamic yield stress where R^2^ = 0.896, and CatBoost gave the highest accuracy for plastic viscosity where R^2^ = 0.941. SHAP and PDP analyses identified total cellulose ether dosage, W/B ratio, silica fume, and sand-to-binder ratio as dominant variables. The optimal blending window was located near a balanced HPMC–HEMC ratio of 0.50, where HPMC = 0.092% and HEMC = 0.092%.

## 1. Introduction

Fresh cement-based materials must maintain a delicate balance between mobility and stability before setting. In practical construction, insufficient fluidity causes poor filling, pumping difficulty, and incomplete compaction, whereas excessive fluidity may induce bleeding, segregation, and nonuniform hardening. For composite cement pastes containing supplementary cementitious materials and chemical admixtures, this balance is governed not only by the water-to-binder ratio and superplasticizer dosage, but also by the rheology-modifying effect of polymeric additives. Among these additives, cellulose ethers are widely used because they improve water retention, increase paste cohesion, and regulate early structural build-up. However, the same thickening and water-retaining capacity that improves stability can also reduce spreadability and increase flow resistance. Therefore, the rational use of cellulose ethers requires a quantitative evaluation of fluidity, dynamic yield stress, and plastic viscosity rather than relying on a single empirical workability index.

Hydroxypropyl methylcellulose (HPMC) and hydroxyethyl methylcellulose (HEMC) are two representative cellulose ethers used in cement-based composites. Although they share similar cellulose backbones, their substituent groups, viscosity grades, dissolution behavior, water-binding ability, and interaction with cement hydration products may lead to different rheological responses. Recent research has confirmed that HPMC and HEMC do not play identical roles in cementitious systems. For Portland cement–calcium sulphoaluminate cement composites, HPMC was reported to produce a more pronounced enhancement in initial viscosity and a faster structural build-up rate than HEMC under comparable conditions [1]. Related work on HPMC-modified Portland cement–sulphoaluminate cement composites further showed that HPMC can increase viscosity, reduce flowability, influence setting time, and alter early hydration and microstructure [2]. For HEMC-modified ultra-high-performance concrete, the content and molecular chain characteristics of HEMC were found to be key factors affecting rheological characteristics and hydration kinetics [3]. These findings indicate that HPMC and HEMC are treated as interchangeable viscosity-modifying admixtures.

The rheological behavior of composite cement paste is also strongly affected by the dispersion–flocculation balance produced by water, superplasticizer, supplementary cementitious materials, and polymers. Mathematical modeling of apparent viscosity has shown that water–cement or water–binder ratio and water-reducing admixture dosage can substantially reshape the viscosity response of cement pastes [4]. In polymer-modified cement composites, rheological enhancement has been linked to changes in yield stress, apparent viscosity, thixotropy, and interparticle interaction forces [5]. In ultra-high-performance fiber-reinforced concrete, rheological evaluation has also demonstrated that superplasticizer dosage and binder composition jointly control the transition between flowability and stability [6]. These studies suggest that cellulose ether modification cannot be interpreted independently from the surrounding mixture system. In particular, HPMC–HEMC blending may produce a response that differs from the simple linear superposition of two single cellulose ethers, because polymer dissolution, water competition, adsorption, and particle bridging may occur simultaneously.

A growing body of research has attempted to improve the design of fresh cement-based materials through rheological testing and mixture optimization. For sprayed ultra-high-performance concrete, the coupling of binder-system design, accelerator dosage, rheology, and hydration kinetics has been used to achieve both early strength and pumpability [7]. For 3D-printed cementitious materials, response surface methodology has been applied to optimize HPMC, accelerator, and polycarboxylate superplasticizer contents with respect to flowability and dynamic yield stress [8]. These studies demonstrate that rheological design is increasingly moving from a single-factor adjustment toward multivariable optimization. Nevertheless, conventional experimental design methods still face several limitations. First, they usually require a large number of trial mixtures when multiple variables and nonlinear interactions are involved. Second, they often provide local response surfaces within a limited design region. Third, they may identify an empirical optimum without revealing the relative contribution of each variable or the interaction mechanism behind the optimum.

Machine learning has recently been introduced into the prediction and optimization of fresh-state properties of cementitious materials, but the available studies differ considerably in dataset size, input variable definition, target outputs, algorithms, and validation depth. Zafar et al. [9] used 77 laboratory samples containing nano-clay, silica fume, bentonite, and methylcellulose to optimize plastic viscosity, dynamic yield stress, and static yield stress of 3D-printable cementitious mixtures through ensemble learning. Their study demonstrated the usefulness of ML for small experimental datasets, but it focused on 3D-printing admixture systems rather than cellulose–ether blending. Tian et al. [10] applied RF, XGBoost, ANN, and stacked LSTM models to predict shear stress–shear rate and apparent viscosity–shear rate curves of cementitious materials containing fly ash and nanosilica, with the stacked LSTM achieving R^2^ values of 0.9582 and 0.9257 for the two curve-prediction tasks. However, cellulose ether dosage and blending ratio were not included as explicit design variables. Navarrete et al. [11] used eight input variables, including supplementary cementitious material properties, cement reactivity, mixture design parameters, and resting time, to predict the time evolution of static yield stress of blended cement pastes, and found that multilayer perceptron gave the most accurate prediction. Nevertheless, the target was static yield stress rather than a combined assessment of fluidity, dynamic yield stress, and plastic viscosity. Chen et al. [12] compiled 380 recycled coarse aggregate concrete mixtures with 11 input features and predicted dynamic yield stress and plastic viscosity using ANN, DT, RF, XGBoost, LightGBM, and SVM. Their results showed that DT achieved the best prediction for dynamic yield stress and XGBoost performed best for plastic viscosity, with test R^2^ values of 0.95 and 0.93, respectively. However, the database addressed recycled aggregate concrete rather than cellulose–ether-modified composite cement paste. These studies confirm the feasibility of ML for rheology prediction, but they also show that previous research generally treated polymer or admixture effects as single input variables, emphasized either one material system or one rheological response family, and rarely decomposed cellulose ether modification into HPMC dosage, HEMC dosage, total cellulose ether dosage, and blending ratio.

However, prediction accuracy alone is insufficient for guiding the design of HPMC–HEMC compound systems. A highly accurate black-box model may predict fluidity or viscosity, but it does not directly explain whether the response is dominated by total cellulose ether dosage, HPMC fraction, HEMC fraction, PCE dosage, water-to-binder ratio, or their interactions. This issue is particularly important for cellulose ether blending, because the design objective is not merely to predict a measured value but to identify a physically meaningful blending window. Recent constitutive modeling of cement paste rheology has emphasized that raw-material composition and solid volume fraction should be incorporated into forward prediction frameworks to improve the interpretability of mixture design [13]. At the same time, fundamental rheological studies have shown that cement paste behaves as a complex particulate suspension whose yield stress can be strongly affected by interparticle contact, shear protocol, and measurement geometry [14,15]. These findings imply that data-driven models should be interpreted cautiously and linked back to material mechanisms.

Despite the progress summarized above, several gaps remain. First, existing cellulose ether studies have mainly focused on single HPMC or single HEMC systems, while a systematic investigation of HPMC–HEMC compound effects on composite cement paste rheology remains limited. Second, most rheological optimization studies emphasize flowability, yield stress, or viscosity separately, whereas the practical design of cement paste requires a coordinated evaluation of all three indicators. Third, machine learning studies on cementitious rheology have generally treated cellulose ether as one input variable, without explicitly decomposing it into HPMC dosage, HEMC dosage, total cellulose ether dosage, and blending ratio. Fourth, although explainable machine learning has been increasingly introduced into construction-material research, its use for identifying the synergistic rheological window of dual cellulose ethers is still insufficient. These gaps restrict the transition from empirical cellulose ether selection to data-driven and mechanism-informed compound admixture design.

Accordingly, the present study focuses on the topic “Synergistic influence of HPMC–HEMC blending on the rheological properties of composite cement paste based on explainable machine learning.” The central objective is to establish a combined experimental and data-driven framework for quantifying how HPMC dosage, HEMC dosage, total cellulose ether dosage, and HPMC–HEMC blending ratio affect fluidity, dynamic yield stress, and plastic viscosity. This study first constructs a controlled mixture design system in which water-to-binder ratio, PCE dosage, HPMC dosage, HEMC dosage, and resting time are recorded as input variables. Flow-table and rheological tests are then used to obtain the target outputs. Based on the cleaned and normalized dataset, several machine learning models, including support vector regression, random forest, XGBoost, CatBoost, and particle-swarm-optimized boosting models, are trained and compared. Finally, SHAP, PDP, interaction analysis, and a synergy index are combined to identify the dominant variables, nonlinear thresholds, and feasible HPMC–HEMC compound window.

The main contributions of this study are summarized as follows. First, a rheology-oriented variable system is established for HPMC–HEMC compound-modified composite cement paste, in which HPMC, HEMC, total cellulose ether dosage, and blending ratio are explicitly represented rather than merged into a single polymer dosage. Second, a multi-output evaluation framework is adopted by considering fluidity, dynamic yield stress, and plastic viscosity together, thereby avoiding the limitation of optimizing only spread diameter or only viscosity. Third, predictive machine learning models are developed and compared to determine the most reliable model for each rheological indicator. Fourth, explainable machine learning methods are introduced to convert model prediction into material-design knowledge, with particular attention to the interaction between HPMC and HEMC. Fifth, a synergy index is proposed to evaluate whether the compound system deviates from the linearly expected response of single HPMC and single HEMC reference systems. Through these contributions, this study aims to provide a quantitative basis for designing cellulose ether compound systems with balanced flowability, stability, and viscosity.

## 2. Materials and Methods

### 2.1. Raw Materials, Mixture Design, and Experimental Variable Setting

The methodological design was established to convert the qualitative regulation of fresh cement-based materials into a quantitatively traceable workflow. Fresh composite cement paste containing cellulose ether is governed by simultaneous effects of particle packing, water film thickness, polymer adsorption, superplasticizer dispersion, and time-dependent structural build-up. These effects are especially difficult to isolate when hydroxypropyl methylcellulose (HPMC) and hydroxyethyl methylcellulose (HEMC) are used together, because both polymers improve water retention and viscosity but may differ in molecular substitution, solution viscosity, adsorption affinity, and steric interaction with polycarboxylate superplasticizer (PCE). Recent studies on fresh cementitious systems have shown that fluidity, dynamic yield stress, and plastic viscosity are more informative than a single consistency index for characterizing workability and construction stability [16,17,18,19]. Therefore, the experimental design was not limited to evaluating whether cellulose ether reduces flowability; instead, it was arranged to determine how the total cellulose ether dosage and the HPMC–HEMC proportion jointly reshape the rheological response surface.

**Figure 1:**
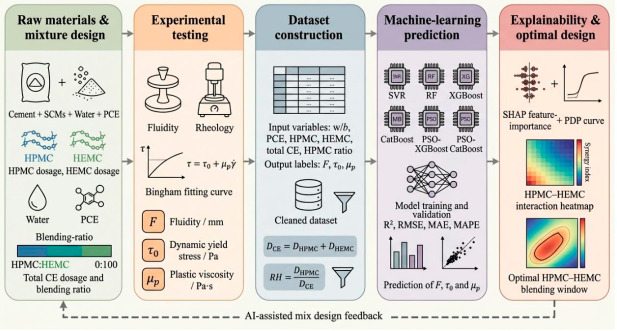
Overall technical route.

The overall logic of this research is summarized in Figure 1, where the sequence starts from raw-material characterization and mixture proportion design, proceeds through fluidity and rheological testing, and then connects the experimental dataset to machine learning prediction and explainable analysis. The diagram is intended to emphasize that the experimental and data-driven parts are not two independent modules. The data structure used for SVR, RF, XGBoost, CatBoost, PSO-XGBoost, and PSO-CatBoost originates directly from the controlled mixture variables, while the subsequent SHAP, PDP, interaction effect, and synergy index analyses are used to return the model outputs to the material-design problem. This arrangement follows the recent machine learning-driven framework for predicting fresh cement-based material workability proposed by Liu et al. [16], but the variable system is expanded from a single HPMC input toward a dual-cellulose ether blending system in which HPMC dosage, HEMC dosage, total cellulose ether dosage, and their blending ratio are treated as explicit design descriptors.

Ordinary Portland cement, supplementary cementitious materials, fine aggregate, PCE, HPMC, HEMC, and mixing water constituted the raw-material system. The chemical and physical information listed in Table 1 provides the necessary basis for interpreting later model features. Oxide composition, density, particle-size index, loss on ignition, and specific surface area determine the water demand and particle interaction of the binder, while the viscosity grade and substitution characteristics of cellulose ether determine the intensity of polymer-induced thickening. Cellulose ether and starch ether have been reported to influence consistency, air content, water retention, and hydration in fresh mortars [20,21], and recent reviews have further indicated that cellulose ether modifies fresh cementitious materials through water retention, adsorption, intermolecular entanglement, and particle-bridging effects [22]. Accordingly, the physical–chemical properties of both HPMC and HEMC are not auxiliary information but the basis for setting rational dosage ranges.

The mixture design was then formulated by separating constant background variables from adjustable experimental variables. The constant background variables ensured the comparability of rheological measurements, whereas the adjustable variables were selected to reveal HPMC–HEMC synergy. The water-to-binder ratio, PCE dosage, total cellulose ether dosage, and HPMC/HEMC mass ratio were therefore taken as the principal control variables. Table 2 presents the recommended variable structure.

The quantitative relationship among the designed variables was defined before model construction so that each mixture could be represented by physically meaningful descriptors rather than by isolated mass values. The water-to-binder ratio was calculated as

(1) $$(w/b)=\frac{m_{w}}{m_{c}+m_{\mathrm{FA}}+{\;m}_{\mathrm{SF}}}$$

where $m_{w}$, $m_{c}$, $m_{\mathrm{FA}}$, and $m_{\mathrm{SF}}$ denote the masses of water, cement, fly ash, and silica fume, respectively. The total cellulose ether dosage was expressed on the basis of binder mass:

(2) $$D_{\mathrm{CE}}\;=\;\frac{m_{\mathrm{HPMC}}\;+\;m_{\mathrm{HEMC}}}{m_{c\;}+\;m_{\mathrm{FA}}\;+\;m_{\mathrm{SF}}}\;\times \;100\%$$

To distinguish between dosage effect and blending-ratio effect, the HPMC fraction within the cellulose ether system was defined as

(3) $$R_{H}=\frac{m_{\mathrm{HPMC}}}{m_{\mathrm{HPMC}}+m_{\mathrm{HEMC}}},\;\;\;R_{E}=1-R_{H}$$

where $R_{E}$ is the corresponding HEMC fraction. The interaction between PCE and cellulose ether was represented by a dosage ratio,

(4) $$\Lambda =\frac{D_{\mathrm{PCE}}}{D_{\mathrm{CE}}+\varepsilon }$$

where ε is a small constant used only to avoid division by zero in mixtures without cellulose ether. This feature is meaningful because the dispersion-thickening balance determines whether a mixture behaves as a highly flowable paste, a stable viscous paste, or a system with insufficient flow despite adequate water content. Similar concerns regarding nonlinear interactions among water, superplasticizer, mineral admixtures, and viscosity-modifying admixtures have been raised in recent work on workability prediction and rheology-oriented mixture design [23,24].

### 2.2. Rheological Properties and Basic Performance Testing

The basic performance testing was designed to provide direct experimental anchors for the machine learning outputs. Fluidity describes the macroscopic spreading capacity of the paste, whereas dynamic yield stress and plastic viscosity describe the stress required to initiate and maintain flow under shear. The simultaneous measurement of these three indicators avoids the limitation that mixtures with similar spread diameters may have different resistance results to pumping, casting, bleeding, or segregation. Recent rheological reviews have emphasized that yield stress and plastic viscosity are fundamental parameters for mixture design, stability assessment, pumping prediction, and 3D-printing-related buildability [25]. Consequently, the flow table and rotational rheometer were combined as complementary rather than alternative tests.

**Figure 2:**
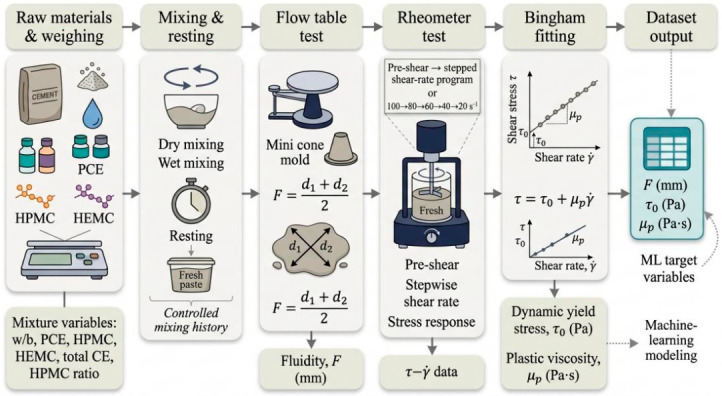
Schematic procedure of fluidity and rheological tests.

Figure 2 organizes the testing process from weighing, dry mixing, wet mixing, resting, flow-table measurement, rheometer loading, pre-shearing, stepwise shear-rate measurement, and Bingham fitting. The flow test was conducted first to minimize structural rebuilding before spread measurement. The rheological test was then conducted under a fixed protocol so that the apparent stress response could be compared across mixtures. The rheometer protocol includes a pre-shear stage to reduce the influence of inconsistent loading history, followed by a descending or stepped shear-rate sequence to obtain stable shear stress values. For each shear-rate level, only the stable part of the stress signal is averaged, because the initial transient interval may reflect thixotropic breakdown rather than steady flow.

The spread diameter was calculated as the arithmetic mean of two perpendicular diameters:

(5) $$F\;=\;\frac{d_{1}\;+\;d_{2}}{2}$$

where $d_{1}$ and $d_{2}$ are the measured diameters after table flow. The rheological behavior of fresh paste was fitted using the Bingham model,

(6) $$\tau =\tau_{0}+{\;\mu }_{p}\dot{\gamma }$$

where τ is shear stress, $\dot{\gamma }$ is shear rate, $\tau_{0}$ is dynamic yield stress, and $\mu_{p}$ is plastic viscosity. When the linearity of the stress–shear-rate curve is acceptable, $\tau_{0}$ and $\mu_{p}$ can be obtained by least-squares fitting:

(7) $$\left({\hat{\tau }}_{0},{\hat{\mu }}_{p}\right)\;=\;\arg\underset{a,b}{\min}\sum_{j=1}^{q}{\left(\tau_{j}-a-b{\dot{\gamma }}_{j}\right)}^{2}$$

The apparent viscosity at a given shear rate was used as a supplementary diagnostic indicator rather than a target variable:

(8) $$\eta_{\mathrm{app}}(\dot{\gamma })\;=\;\frac{\tau }{\dot{\gamma }}$$

These equations support the chain from physical testing to dataset construction. Equation (5) provides the experimental label for flowability; Equations (6) and (7) convert rheometer signals into dynamic yield stress and plastic viscosity; Equation (8) helps identify abnormal stress responses caused by wall slip, local segregation, or incomplete homogenization.

To guarantee reproducibility, each mixture is tested at least three times when material availability permits. The coefficient of variation was used to determine whether repeated measurements were acceptable:

(9) $$\mathrm{CV}=\frac{s}{\bar{x}}\times 100\%$$

If the CV of fluidity, dynamic yield stress, or plastic viscosity exceeds a preset threshold, the mixture is rechecked for weighing error, mixing inconsistency, air entrainment, or early stiffening. Such quality control is important because machine learning models can fit noisy data but cannot distinguish physical nonlinearity from experimental error without proper preprocessing.

### 2.3. Dataset Construction and Preprocessing

The dataset was constructed by combining mixture descriptors, raw-material descriptors, test-condition descriptors, and measured output labels. For the i-th sample, the input vector was defined as

(10) $$x_{i}=[w/b,D_{\mathrm{HPMC}},D_{\mathrm{HEMC}},D_{\mathrm{CE}},R_{H},t_{r},\ldots ]$$

and the output vector was defined as

(11) $$y_{i}={\left[F,\tau_{0},\mu_{p}\right]}_{i}$$

This representation retains both the original dosage information and derived physical descriptors. The reason is that tree-based models can use original variables efficiently, while kernel-based models such as SVR often benefit from normalized and well-scaled descriptors. Moreover, including both $D_{\mathrm{CE}}$ and $R_{H}$ makes it possible to distinguish whether a predicted change in viscosity is caused by the overall polymer amount or by the substitution of HPMC with HEMC [26].

Before model training, missing values, duplicated records, abnormal values, and inconsistent units were processed according to Algorithm 1. Algorithm 1 summarizes the complete data preprocessing procedure, including unit standardization, missing-value checking, duplicate removal, outlier screening, data splitting, and feature normalization. Missing values for indispensable output labels were not imputed, because artificial target values would distort model evaluation. Missing values in auxiliary input variables were handled only when the corresponding information could be recovered from mixture proportions or material reports. Outlier identification was based on standardized distance and physical plausibility, not on statistical thresholds alone. The standardized value of each feature was computed as

(12) $$z_{\mathrm{ij}}=\frac{x_{\mathrm{ij}}-\mu_{j}}{\sigma_{j}}$$

where $\mu_{j}$ and $\sigma_{j}$ are the mean and standard deviation of feature j in the training set. A candidate outlier was flagged when

(13) $$z_{\mathrm{ij}}\;>\;3$$

However, a flagged record was removed only if it contradicted material logic, testing records, or replicate consistency. This conservative strategy is necessary because high-yield-stress or high-viscosity mixtures may be physically valid, especially at a high cellulose ether dosage.

The cleaned dataset was randomly divided into training and testing subsets at a ratio of 80:20 using a fixed random seed, resulting in 180 samples for model training and 45 samples for independent testing. To avoid a favorable or biased random division, the statistical representativeness of the two subsets was examined before model training. The distributions of the input and output variables were compared using descriptive statistics, standardized mean difference, and the two-sample Kolmogorov–Smirnov test. The standardized mean difference was calculated as the absolute difference between the training and testing means divided by the pooled standard deviation. For the 11 input variables, the standardized mean differences ranged from 0.018 to 0.176, and the Kolmogorov–Smirnov test *p*-values ranged from 0.213 to 0.984. For the three output variables, the standardized mean differences ranged from 0.030 to 0.071, and the *p*-values ranged from 0.584 to 0.912. Specifically, the mean fluidity values of the training and testing subsets were 138.2 and 136.8 mm, respectively; the mean dynamic yield stress values were 1160.8 and 1171.9 Pa, respectively; and the mean plastic viscosity values were 9.46 and 9.57 Pa·s, respectively. All *p*-values were higher than 0.05, indicating that no statistically significant distributional difference existed between the training and testing subsets. Therefore, the data split was considered statistically representative and suitable for subsequent model training and independent performance evaluation.
**Algorithm 1** Data preprocessing procedure for machine learning modeling1Import raw mixture table, raw-material table, and testing-result table.2Convert all dosage variables to binder-mass percentage and all rheological outputs to unified units.3Remove duplicated records and records lacking indispensable output labels.4Derive D_CE_, R_H_, R_E_, and Λ using Equations (2)–(4).5For each feature and each output, compute descriptive statistics and standardized values using Equation (12).6Flag records satisfying Equation (13) as candidate outliers.7Retain physically valid extreme records and remove only confirmed erroneous records.8Split the cleaned dataset into training and testing subsets using a fixed random seed.9Fit normalization parameters on the training set only and apply them to both training and testing subsets.10Export the cleaned input matrix X and output vectors F, τ_0_, and μ_p_.

### 2.4. Machine Learning Modeling and Model Optimization

The modeling strategy was designed to compare algorithms with different bias–variance characteristics and different capacities for capturing nonlinear relationships in tabular mixture datasets. In this study, six regression models were considered, namely support vector regression (SVR), random forest (RF), extreme gradient boosting (XGBoost), categorical boosting (CatBoost), particle-swarm-optimized XGBoost (PSO-XGBoost), and particle-swarm-optimized CatBoost (PSO-CatBoost). For each rheological output, including fluidity, dynamic yield stress, and plastic viscosity, a separate regression model was trained and evaluated. Based on the statistically representative 80:20 data split described in Section 2.3, each model was trained on the training subset and independently evaluated on the testing subset [27].

SVR was used as a kernel-based regression model. It maps the input variables into a high-dimensional feature space and constructs an optimal regression function by minimizing the prediction error within a predefined insensitive-loss margin. In this study, the radial basis function kernel was adopted because it is suitable for nonlinear material-property prediction problems. The main SVR hyperparameters include the penalty coefficient C, the kernel coefficient γ, and the insensitive-loss parameter ε. RF was used as a bagging-based ensemble model. It constructs multiple decision trees using bootstrap samples from the training dataset and randomly selected feature subsets at each split. The final prediction is obtained by averaging the outputs of all trees. This strategy reduces variance and improves robustness against noise and overfitting. The main RF hyperparameters include the number of trees, maximum tree depth, minimum number of samples required for splitting, and minimum number of samples in each leaf node. XGBoost was selected as a gradient-boosting decision-tree model. Unlike RF, which trains trees independently, XGBoost builds trees sequentially, and each new tree is trained to correct the residual errors of the previous ensemble. The objective function includes both a loss term and a regularization term, which helps improve prediction accuracy while controlling model complexity. The main XGBoost hyperparameters include the number of estimators, learning rate, maximum tree depth, subsampling ratio, column-sampling ratio, and regularization coefficients. CatBoost was also used as a gradient-boosting model. It applies ordered boosting and symmetric decision trees to reduce prediction shift and improve generalization performance. Although the input variables in this study are mainly numerical, CatBoost was included because of its strong performance on small- and medium-sized tabular datasets and its robustness to complex nonlinear feature interactions. The main CatBoost hyperparameters include the number of iterations, learning rate, tree depth, L2 leaf regularization coefficient, and loss function [28,29,30,31,32].

To further improve the performance of boosting models, PSO was used to optimize the hyperparameters of XGBoost and CatBoost. In PSO-XGBoost and PSO-CatBoost, each particle represents a candidate hyperparameter combination. The model performance on the validation subset was used as the fitness value, and the particle positions were updated iteratively according to the personal best and global best solutions. After the PSO search process, the optimal hyperparameter combination was used to retrain the corresponding model on the training dataset. This procedure was adopted to reduce the subjectivity of manual parameter selection and to improve the reproducibility of model optimization.

The final optimized hyperparameters were recorded to improve model reproducibility. For SVR, the RBF kernel was adopted, and the optimized ranges of C, gamma, and epsilon were 85–120, 0.038–0.050, and 0.06–0.10, respectively, depending on the target variable. For RF, the optimized settings included a range of 500–600 trees, a maximum depth range of 8–10, a minimum split size of 2, a minimum leaf size of 1, and the square-root feature-selection strategy. For XGBoost, the optimized ranges of the number of estimators, learning rate, maximum depth, subsampling ratio, column-sampling ratio, and L2 regularization coefficient were 420–520, 0.030–0.035, 4–5, 0.82–0.86, 0.88–0.90, and 1.0–1.5, respectively. For CatBoost, the optimized ranges of iterations, learning rate, tree depth, and L2 leaf regularization coefficient were 650–720, 0.025–0.030, 5–6, and 3.0–4.0, respectively, with RMSE used as the loss function. For PSO-XGBoost and PSO-CatBoost, PSO was used to optimize the corresponding tree number or iteration number, learning rate, tree depth, subsampling-related parameters, and regularization coefficients. The final PSO-optimized ranges were 460–560 estimators, a learning rate of 0.024–0.028, a maximum depth of 4–5, a subsampling ratio of 0.84–0.88, a column-sampling ratio of 0.86–0.88, and an L2 regularization coefficient of 1.2–1.7 for PSO-XGBoost. For PSO-CatBoost, the optimized ranges were 720–800 iterations, a learning rate of 0.022–0.026, a tree depth of 5–6, and an L2 leaf regularization coefficient of 3.5–4.2. These optimized hyperparameters were fixed during the final model training and testing stage.

For each output variable, a separate regression model was trained. This single-output strategy was adopted because fluidity, dynamic yield stress, and plastic viscosity may have different dominant variables and different nonlinear response patterns. However, this target-specific modeling strategy also has limitations. Because fluidity, dynamic yield stress, and plastic viscosity were predicted by separate regression models, the intrinsic correlations among the three rheological outputs were not directly constrained during model training. As a result, the independently predicted outputs may theoretically produce combinations that are less physically consistent for a given mixture, especially outside the experimental design space. Therefore, the present single-output modeling framework is regarded as a practical predictive strategy for identifying target-specific dominant variables rather than a fully coupled multi-output rheological model. In future work, multi-output regression models and deep-learning architectures will be considered to capture the coupled relationships among fluidity, dynamic yield stress, and plastic viscosity more explicitly. Model performance was assessed using the coefficient of determination, root mean square error, and mean absolute error:

(14) $$R^{2}\;=\;1-\frac{\sum_{i=1}^{n}{\left(y_{i}-{\hat{y}}_{i}\right)}^{2}}{\sum_{i=1}^{n}{\left(y_{i}-\bar{y}\right)}^{2}},$$

(15) $$\mathrm{RMSE}=\sqrt{\frac{1}{n}\sum_{i=1}^{n}{\left(y_{i}-{\hat{y}}_{i}\right)}^{2}},$$

(16) $$\mathrm{MAE}=\frac{1}{n}\sum_{i=1}^{n}\left|y_{i}-{\hat{y}}_{i}\right|.$$

These metrics complement each other: $R^{2}$ reflects explained variance, RMSE penalizes large errors, and MAE measures average absolute deviation. For SVR, the regression function was expressed as

(17) $$\hat{y}(x)=\sum_{i=1}^{n}\left(\alpha_{i}-\alpha_{i}^{*}\right)K\left(x_{i},x\right)+b,$$

where K(⋅) is the kernel function, $\alpha_{i}$ and $\alpha_{i}^{*}$ are Lagrange multipliers, and b is the intercept. For PSO, each particle represented a candidate hyperparameter vector. Its velocity and position were updated by

(18) $$v_{k}^{t+1}=\omega v_{k}^{t}+c_{1}r_{1}\left(p_{k}^{t}-s_{k}^{t}\right)+c_{2}r_{2}\left(g^{t}-s_{k}^{t}\right),\;\;\;s_{k}^{t+1}=s_{k}^{t}+{\;v}_{k}^{t+1}$$

where $s_{k}^{t}$ and $v_{k}^{t}$ denote the position and velocity of particle k at iteration t, $p_{k}^{t}$ is the personal best position, $g^{t}$ is the global best position, ω is the inertia weight, and $c_{1}$ and $c_{2}$ are acceleration coefficients.

Model selection was not based solely on the highest testing R^2^. Residual distribution, error concentration at high-viscosity mixtures, and prediction stability under cross-validation were considered together. This requirement is essential because the practical objective is to identify a reliable HPMC–HEMC blending window rather than to maximize a statistical score. A model that performs well on average but systematically underestimates high yield stress would be unsuitable for mixture design because it may incorrectly classify a stiff mixture as pumpable or castable.

### 2.5. Explainable Analysis and Synergy Index Calculation

The purpose of explainable machine learning was to translate the predictive model into material-design knowledge. Feature importance alone is insufficient for this task because HPMC dosage, HEMC dosage, total cellulose ether dosage, and blending ratio are mathematically related and physically coupled. Therefore, SHAP, PDP, SHAP interaction values, and a separately defined synergy index were combined. This combination allows three levels of interpretation: global ranking of variables, marginal response trends, and pairwise interaction or blending synergy. SHAP and PDP have been used in recent fresh cement-based material prediction studies to reveal the contribution of mixture components to fluidity and rheological outputs, while broader interpretable machine learning literature emphasizes that interpretation methods are used to infer model-learned relationships rather than to claim causality without experimental support [33,34].

Explainable machine learning was used to bridge predictive modeling and material interpretation. Instead of treating the trained models as purely numerical tools, SHAP, PDP, and feature-interaction analysis were adopted to quantify the contribution of each input variable, identify nonlinear thresholds, and reveal whether two variables jointly amplified or weakened a target response. For HPMC–HEMC compound modification, this analysis is particularly important because the same total cellulose ether dosage may produce different rheological behavior when the HPMC/HEMC ratio changes, while the same HPMC/HEMC ratio may also have different effects under different water-to-binder ratios or PCE dosages. Therefore, the explainable analysis was used to distinguish the dosage effect from the blending-ratio effect; identify the dominant variables for fluidity, dynamic yield stress, and plastic viscosity; and provide evidence for evaluating whether HPMC and HEMC exhibited synergistic or antagonistic behavior.

For a trained model f, SHAP decomposes the prediction of sample i as

(19) $${\hat{y}}_{i}=f\left(x_{i}\right)=\phi_{0}+\sum_{j=1}^{p}\phi_{ij}$$

where ${\hat{y}}_{i}$ is the predicted output of sample i, $x_{i}$ is the input vector of sample i, *p* is the number of input variables, $\phi_{0}$ is the expected model output, and $\phi_{ij}$ is the SHAP contribution of feature j to the prediction of sample i. The global importance of feature j was calculated as the mean absolute SHAP value over all samples.

(20) $${\mathrm{PDP}}_{j}(z)=\frac{1}{n}\sum_{i=1}^{n}f\left(z,x_{i,-j}\right),$$

where z is a specified value of feature $x_{i,-j}$ denotes all features of sample i except $x_{j}$, and *n* is the number of samples used for PDP calculation. This function describes the marginal effect of feature $x_{j}$ on the predicted response after averaging over the empirical distribution of the remaining variables.

To evaluate pairwise feature interactions, the model prediction was further decomposed using SHAP interaction values:

(21) $${\hat{y}}_{i}=f\left(x_{i}\right)=\phi_{0}+\sum_{j=1}^{p}\phi_{ij}+\sum_{j<k}\phi_{ijk}^{int}$$

where $\phi_{ijk}^{int}$ represents the interaction contribution between features *j* and *k* for sample *i*. In this study, particular attention was given to the interaction between HPMC dosage and HEMC dosage, because this interaction reflects whether the two cellulose ethers act independently or produce a coupled rheological response.

A synergy index was further introduced to quantify whether the actual response of an HPMC–HEMC blend deviates from the linearly expected response between the two single-cellulose ether reference systems. For a target property $Y\in \{F,\tau_{0},\mu_{p}\}$ at the same total cellulose ether dosage, the expected linear response was defined as

(22) $$Y_{\mathrm{lin}}\left(R_{H}\right)=R_{H}Y_{\mathrm{HPMC}}+\left(1-R_{H}\right)Y_{\mathrm{HEMC}},$$

where $Y_{\mathrm{HPMC}}$ and $Y_{\mathrm{HEMC}}$ are the responses of the single-HPMC and single-HEMC reference mixtures. The normalized synergy index was calculated as

(23) $$S_{Y}=\frac{Y_{\mathrm{blcnd}}-Y_{\mathrm{lin}}}{\left|Y_{\mathrm{lin}}\right|+\varepsilon }$$

A positive $S_{Y}$ indicates that the blend produces a higher response than linear interpolation, while a negative value indicates an antagonistic or dilution effect. The sign must be interpreted according to the target property. For example, a positive $S_{\tau_{0}}$ or $S_{\mu_{p}}$ may indicate enhanced thickening, whereas a strongly negative $S_{F}$ may indicate excessive loss of flowability. The optimal blending window was therefore identified not by maximizing a single response but by satisfying a multi-objective condition:

(24) $$\Omega^{*}=\left\{x:F_{\min}\leq \hat{F}(x)\leq F_{\max},\tau_{0,\min}\leq {\hat{\tau }}_{0}(x)\leq \tau_{0,\max},\mu_{p,\min}\leq {\hat{\mu }}_{p}(x)\leq \mu_{p,\max}\right\}$$

In the present study, Equation (24) was implemented using a constrained grid-search optimization strategy based on the trained ML surrogate models, rather than by using PSO or gradient-based optimization. PSO was only used for hyperparameter optimization of XGBoost and CatBoost. For property optimization, the HPMC–HEMC design space was discretized by varying the total cellulose ether dosage and the HPMC fraction within their experimental ranges. For each candidate mixture, HPMC dosage, HEMC dosage, total cellulose ether dosage, HPMC ratio, and synergy index were calculated and then combined with the remaining mixture descriptors to form a complete input vector. This vector was passed into the selected best-performing ML models to predict fluidity, dynamic yield stress, and plastic viscosity. The candidate mixture was retained only when all three predicted responses simultaneously satisfied the constraints defined in Equation (24). Therefore, the ML models served as surrogate predictors inside the optimization loop, while Equation (24) served as the feasibility filter for identifying the acceptable HPMC–HEMC blending region.

This final equation links the preceding methods to the material-design objective. Fluidity prevents excessive stiffening, dynamic yield stress prevents instability and segregation, and plastic viscosity controls deformation resistance during continuous flow. The SHAP and PDP results identify which variables move a mixture toward or away from this feasible region, while the synergy index reveals whether HPMC–HEMC blending offers a benefit beyond simple dosage adjustment.

Algorithm 2 shows an interpretable machine learning workflow that takes optimized predictive models for cement paste fluidity, dynamic yield stress, and plastic viscosity as inputs, and iteratively conducts multi-dimensional interpretive analysis for each rheological property by calculating SHAP values to quantify feature contributions and rank variable importance, generating SHAP dependence plots and PDP curves to visualize the nonlinear marginal effects of key HPMC/HEMC formulation parameters, and computing SHAP interaction values and normalized synergy indices to identify coupled interactions and synergistic/antagonistic behaviors between blending components; finally, it adopts a multi-objective constraint strategy to screen feasible HPMC–HEMC mixture formulations that meet all rheological performance requirements and outputs the optimal blending range and corresponding material mechanism interpretation.
**Algorithm 2** SHAP, PDP, interaction-effect, and synergy-index calculation1Input optimal models for F, τ_0_, and μ_p_.2**for** each target output Y **do**3   Compute SHAP values using Equation (19).4   Rank features by mean absolute SHAP value.5   Generate SHAP dependence plots for D_HPMC_, D_HEMC_, D_CE_, and R_H_.6   Calculate PDP curves for key variables using Equation (20).7   Compute SHAP interaction values for HPMC–HEMC and PCE–cellulose-ether feature pairs using Equation (21).8   Calculate $Y_{\mathrm{lin}}$ and $S_{Y}$ using Equations (22) and (23).9**end for**10Identify feasible mixtures satisfying Equation (24).11Output the HPMC–HEMC optimal blending range and corresponding design interpretation.

Through this methodological chain, the experimental design, rheological testing, machine learning modeling, and explainable analysis form a closed loop. The raw-material table defines the physical basis of the system; the mixture-variable table defines the controllable design space; Figure 1 clarifies the overall data flow; Figure 2 standardizes the path from fresh paste preparation to quantitative labels; the preprocessing algorithm protects the dataset from unit errors, outliers, and leakage; the modeling algorithm evaluates predictive reliability; and the explainability algorithm converts the optimal model into a HPMC–HEMC blending rule. This structure is consistent with recent data-driven studies on fresh cement-based materials, but it further introduces explicit blending-ratio descriptors and a synergy index so that the final output is not only a prediction model but also a design-oriented interpretation of cellulose ether compound modification.

## 3. Results and Discussion

### 3.1. Dataset Characteristics and Variable Correlation Analysis

**Figure 3:**
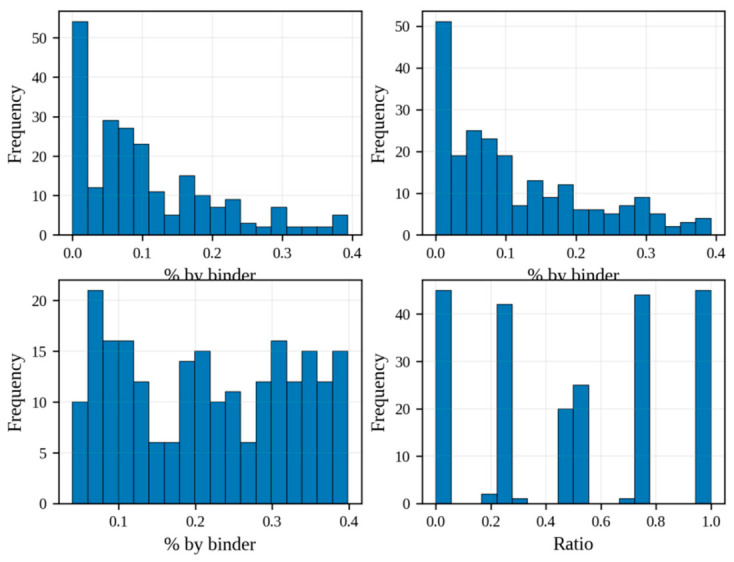
Data distributions of HPMC dosage, HEMC dosage, total cellulose ether dosage, and blending ratio.

The dataset contains 225 mixtures and 11 input variables, including fly ash content, silica fume content, W/B ratio, PCE dosage, sand-to-binder ratio, HPMC dosage, HEMC dosage, total cellulose ether dosage, HPMC ratio, HEMC ratio, and synergy index. The cellulose ether variables cover a broad design space: HPMC ranges from 0 to 0.394% by binder, HEMC ranges from 0 to 0.393%, and total cellulose ether dosage ranges from 0.041 to 0.398%. The distribution in Figure 3 indicates that the dataset includes HEMC-rich, HPMC-rich, and balanced HPMC–HEMC systems, with an average HPMC ratio of 0.500 and an average synergy index of 0.510. This balanced sampling is important because recent studies have shown that HPMC and HEMC may contribute differently to early structural build-up and water absorption in cementitious systems.

**Figure 4:**
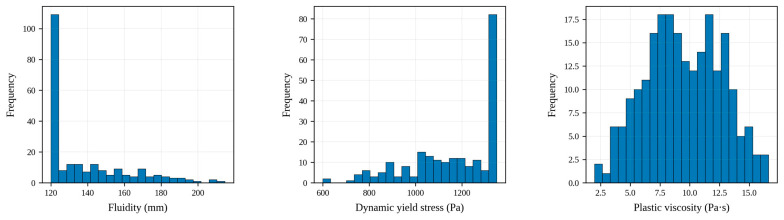
Distribution of test results for flowability, dynamic yield stress, and plastic viscosity.

The response variables also show sufficient variation for machine learning analysis. Fluidity varies from 120.0 to 214.7 mm, with a mean value of 137.9 mm; dynamic yield stress ranges from 599.4 to 1350.0 Pa, with a mean value of 1163.0 Pa; and plastic viscosity ranges from 2.00 to 16.62 Pa·s, with a mean value of 9.48 Pa·s. As presented in Figure 4, fluidity is concentrated mainly in the low-to-medium workability range, whereas yield stress and viscosity show wider dispersion. This distribution is suitable for studying low-flow and high-cohesion cement pastes, which are commonly required in extrusion, repair mortar, and printable cementitious materials. Similar emphasis on flowability, dynamic yield stress, and viscosity has also been reported in recent machine learning-based optimization studies for printable cementitious mixtures.

**Figure 5:**
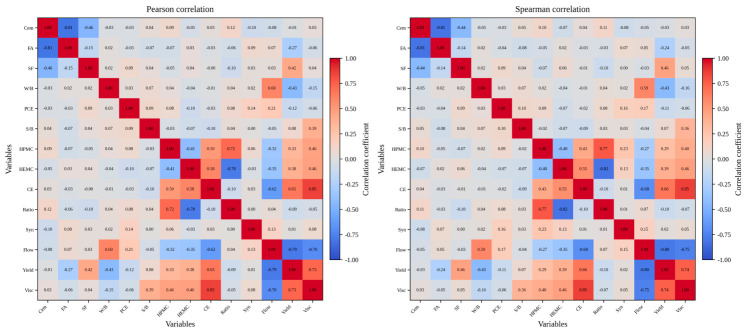
Pearson/Spearman correlation heatmap between input variables and output variables.

The correlation results reveal clear rheological relationships among the variables. In Figure 5, total cellulose ether dosage shows a strong negative Pearson correlation with fluidity, with a coefficient of −0.618, while its correlation with dynamic yield stress and plastic viscosity reaches 0.653 and 0.848, respectively. W/B ratio has the opposite effect: it is positively correlated with fluidity, with a Pearson coefficient of 0.597, but negatively correlated with yield stress and viscosity. The three output variables are also strongly coupled. Fluidity is negatively correlated with yield stress and viscosity, with Pearson coefficients of −0.785 and −0.696, while yield stress and viscosity are positively correlated, with a coefficient of 0.726. The Spearman correlation matrix gives similar trends, confirming that these relationships are not only linear but also monotonic.

**Figure 6:**
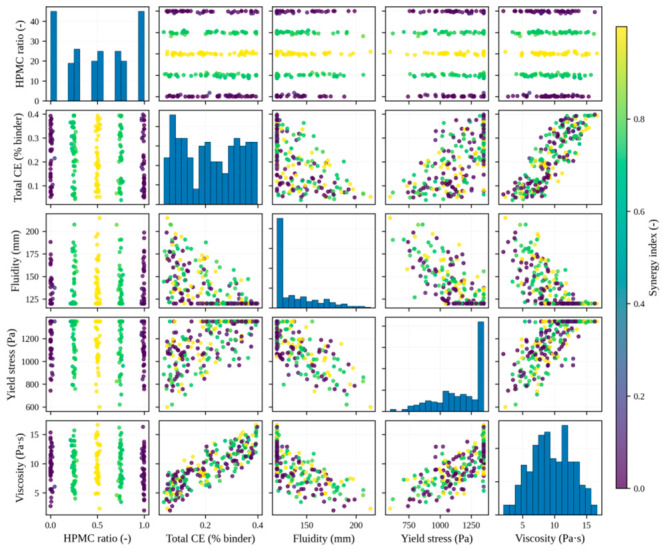
Two-dimensional scatter matrix of HPMC–HEMC blending ratio and rheological properties.

The scatter matrix in Figure 6 further shows that increasing total cellulose ether generally shifts the mixtures toward lower fluidity and higher viscosity. However, the influence of HPMC ratio alone is weaker than that of total cellulose ether dosage, indicating that the rheological response is controlled by both dosage and blending proportion. The balanced HPMC–HEMC region does not simply follow a linear interpolation between pure HPMC and pure HEMC systems, suggesting that the combined network formation, water retention, and particle flocculation effects are analyzed through nonlinear models rather than single-factor regression [35,36].

### 3.2. Effects of HPMC–HEMC Blending on the Rheological Properties of Composite Cement Paste

**Figure 7:**
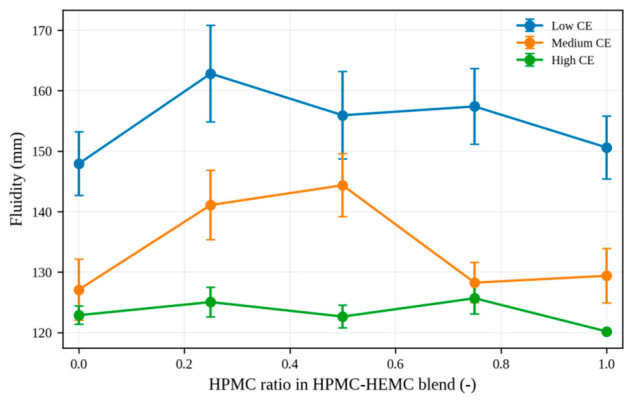
Variation in flowability under different HPMC–HEMC blending ratios.

To clarify the cellulose ether dosage classification used in Figure 7, the mixtures were divided into three groups according to the total cellulose ether dosage D_CE_. Based on the dosage range of the present dataset, Low CE, Medium CE, and High CE correspond to 0.041% ≤ D_CE_ < 0.150%, 0.150% ≤ D_CE_ < 0.300%, and 0.300% ≤ D_CE_ ≤ 0.398% by binder mass, respectively. The influence of HPMC–HEMC blending is first reflected in fluidity. According to Figure 7, HEMC-rich mixtures have an average fluidity of 133.45 mm, whereas the balanced-HEMC and balanced-HPMC groups increase to 141.89 and 141.02 mm, respectively. The HPMC-rich group decreases slightly to 136.89 mm. This indicates that a moderate HPMC–HEMC combination can maintain better flowability than highly HEMC-dominated or highly HPMC-dominated systems. The trend is consistent with the understanding that cellulose ethers improve water retention and cohesion but may reduce flowability when their dosage or thickening effect becomes excessive. Recent experimental work on HPMC-modified Portland cement–sulphoaluminate composites also reported that HPMC increases viscosity while reducing flowability.

**Figure 8:**
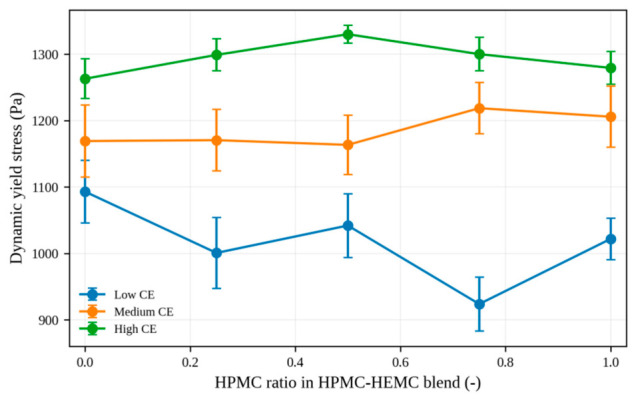
Variation in dynamic yield stress under different HPMC–HEMC blending ratios.

The yield stress response presents a more gradual change with blending proportion. Figure 8 shows that the HEMC-rich group has the highest average dynamic yield stress, 1188.19 Pa, followed by balanced-HEMC mixtures at 1171.23 Pa, balanced-HPMC mixtures at 1151.70 Pa, and HPMC-rich mixtures at 1141.16 Pa. Although the difference among groups is not extremely large, the overall trend suggests that increasing the HPMC fraction slightly reduces the dynamic yield stress under the composition range. This behavior may be related to the different water absorption and structural build-up rates of HPMC and HEMC, because recent PC–CSA composite research reported that HPMC-modified pastes showed more obvious time-dependent yield stress growth than HEMC-modified pastes, while HEMC displayed weaker early yield stress development within the first 60 min.

**Figure 9:**
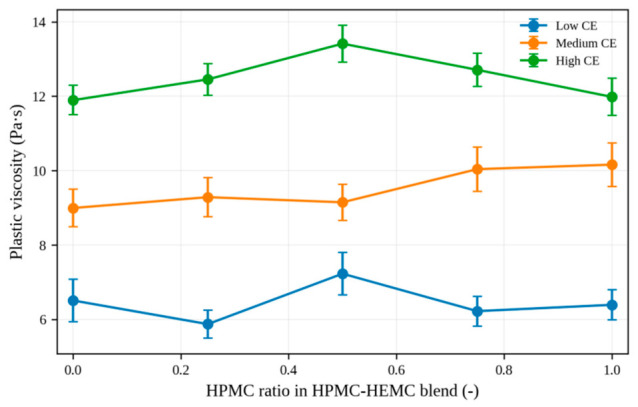
Variation in plastic viscosity under different HPMC–HEMC blending ratios.

The error bars in Figure 7, Figure 8 and Figure 9 represent the standard error of the grouped responses, calculated from the standard deviation divided by the square root of the number of mixtures in each group. Therefore, they describe the dispersion of rheological responses within the same cellulose ether dosage level and HPMC–HEMC blending-ratio group, rather than the uncertainty of the machine learning predictions. The variability mainly originates from experimental and material-related factors, including local differences in mixture composition, water-to-binder ratio, PCE dosage, sand-to-binder ratio, mineral-admixture content, mixing uniformity, resting time, and the inherent sensitivity of fresh-paste rheological measurements. Thus, the error bars primarily reflect aleatoric variability and within-group formulation variability. They do not explicitly quantify epistemic uncertainty caused by model limitations, which is instead evaluated indirectly through model residuals and prediction errors in Section 3.3.

**Figure 10:**
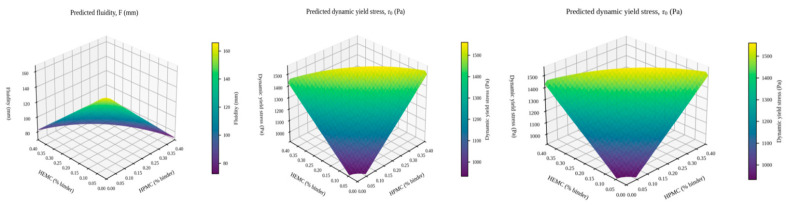
Synergistic response surface of flowability, yield stress, and plastic viscosity in the HPMC–HEMC blended system.

Plastic viscosity is more sensitive to the combined effect of total cellulose ether dosage and blending ratio. In Figure 9, the average viscosity of the HEMC-rich group is 9.67 Pa·s, while the balanced-HPMC group reaches 9.81 Pa·s, which is the highest among the four groups. The HPMC-rich group shows a lower average value of 9.10 Pa·s. This suggests that viscosity enhancement is not maximized by one cellulose ether alone, but by a suitable balance between HPMC and HEMC. The response surfaces in Figure 10 confirm this nonlinearity: as both HPMC and HEMC increase together, the predicted fluidity decreases, whereas yield stress and viscosity increase. In each response surface, the x- and y-axes represent HPMC and HEMC dosages, respectively, while the color scale represents the corresponding predicted model response, namely fluidity F (mm), dynamic yield stress τ_0_ (Pa), or plastic viscosity μp (Pa·s). The strongest interaction region appears near the medium-to-high combined dosage zone, where the synergy index becomes more active [37].

**Figure 11:**
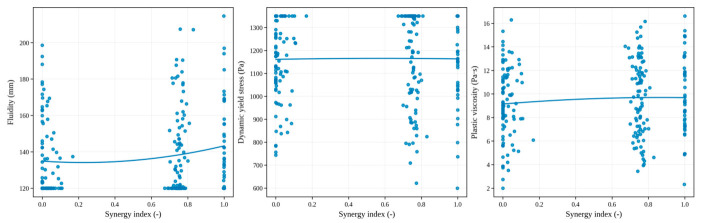
Effect of the HPMC–HEMC synergy index on rheological properties.

**Figure 12:**
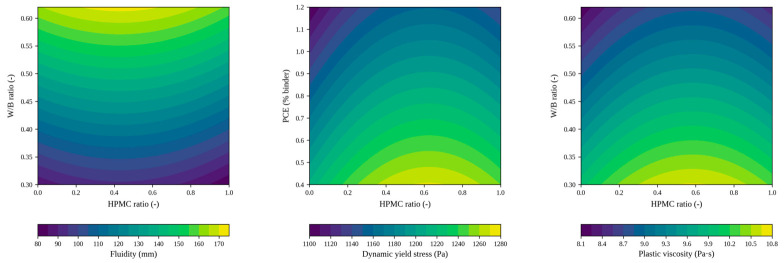
Interaction effects of PCE dosage, water-to-binder ratio, and HPMC–HEMC blending ratio.

The synergy index provides a direct way to identify whether the HPMC–HEMC blend behaves as a simple additive system or as a coupled rheology-modifying system. In Figure 11, the interaction effect reaches approximately 14.14 mm for fluidity, 74.40 Pa for dynamic yield stress, and 0.992 Pa·s for plastic viscosity at a balanced high-dosage condition around HPMC = 0.207% and HEMC = 0.207%. This means that the blend ratio affects not only the magnitude of each rheological parameter but also the coupling among flow resistance, structural build-up, and viscosity. Figure 12 further shows that PCE and W/B ratio can partially offset the thickening effect of cellulose ethers. At a higher W/B ratio and PCE dosage, the system tends to recover fluidity and reduce yield stress, but excessive compensation may narrow the stable rheological window [38].

### 3.3. Comparison of Machine Learning Model Prediction Performance

**Figure 13:**
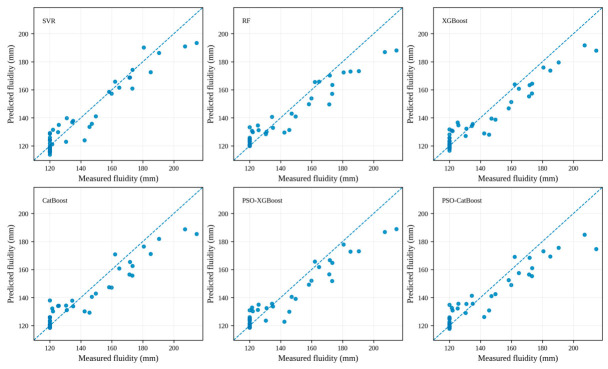
Comparison between predicted and measured flowability values for different models.

The prediction results show that the three rheological targets require different optimal models. For fluidity prediction, Figure 13 shows that the RBF-kernel SVR model provides the closest agreement between measured and predicted values, with a test R^2^ of 0.918, RMSE of 7.60 mm, MAE of 5.84 mm, and MAPE of 4.03%. The radial basis function kernel was selected because it can capture smooth nonlinear relationships between mixture variables and rheological responses, which is consistent with the nonlinear influence of cellulose ether dosage, W/B ratio, and PCE dosage on flowability. XGBoost and RF also perform reasonably well, with test R^2^ values of 0.891 and 0.886, respectively, but their scatter is slightly wider than that of SVR. This result indicates that the relationship between mixture parameters and fluidity is nonlinear but relatively smooth, making SVR effective for capturing the response surface in a medium-sized dataset [39].

**Figure 14:**
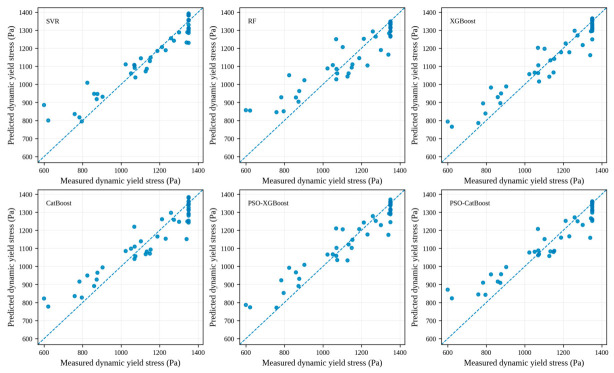
Comparison between predicted and measured dynamic yield stress values for different models.

For dynamic yield stress, Figure 14 shows that XGBoost gives the best overall prediction accuracy, with a test R^2^ of 0.896, RMSE of 71.74 Pa, MAE of 52.17 Pa, and MAPE of 5.40%. SVR and PSO-XGBoost follow closely, with test R^2^ values of 0.891 and 0.890. Compared with fluidity, the yield stress data show stronger dispersion because the output is affected by cellulose ether dosage, silica fume content, W/B ratio, and interaction effects. The good performance of boosting models agrees with recent interpretable machine learning studies on self-compacting concrete, where ensemble models were used to connect mixture proportions with rheological and workability parameters.

The relatively large scatter observed near the lower fluidity boundary of approximately 120 mm is mainly related to the low-flow and high-cohesion region of the dataset. In this region, mixtures with different combinations of high cellulose ether dosage, low W/B ratio, higher silica fume content, and different PCE dosages may produce similarly low measured spread values. Therefore, the measured fluidity becomes less sensitive to compositional differences once the paste approaches the low-flow boundary, whereas the ML model still responds to variations in the input variables. This leads to a wider prediction dispersion around 120 mm. In contrast, at higher fluidity levels, the paste remains more mobile and the relationship between mixture variables and spread diameter is smoother, so the prediction scatter becomes smaller. A similar phenomenon appears in the high dynamic-yield-stress region, around a range of 1300–1400 Pa. This region corresponds to stiff mixtures with high cellulose ether dosage and strong particle flocculation, where small changes in the W/B ratio, silica fume content, PCE dosage, or HPMC–HEMC proportion can cause relatively large changes in yield stress.

**Figure 15:**
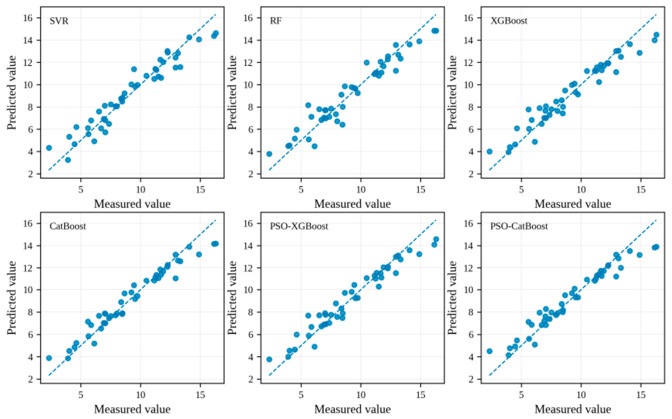
Comparison between predicted and measured plastic viscosity values for different models.

**Figure 16:**
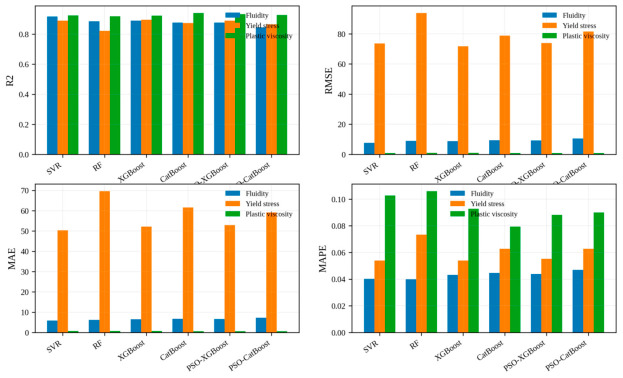
Comparison of evaluation metrics for SVR, RF, XGBoost, CatBoost, PSO-XGBoost, and PSO-CatBoost models.

For plastic viscosity, Figure 15 indicates that CatBoost achieves the highest accuracy, with a test R^2^ of 0.941, RMSE of 0.816 Pa·s, MAE of 0.595 Pa·s, and MAPE of 7.94%. PSO-XGBoost and PSO-CatBoost also perform well, with test R^2^ values of 0.934 and 0.928. The model comparison in Figure 16 confirms that no single model dominates all three outputs: SVR is most suitable for fluidity, XGBoost for dynamic yield stress, and CatBoost for plastic viscosity. This supports the use of target-specific model selection rather than applying one fixed algorithm to all rheological indicators. Recent studies on SCC rheology also showed that SHAP- and PDP-assisted ensemble models can achieve high prediction accuracy when the target response is governed by multiple interacting composition variables [40,41].

**Figure 17:**
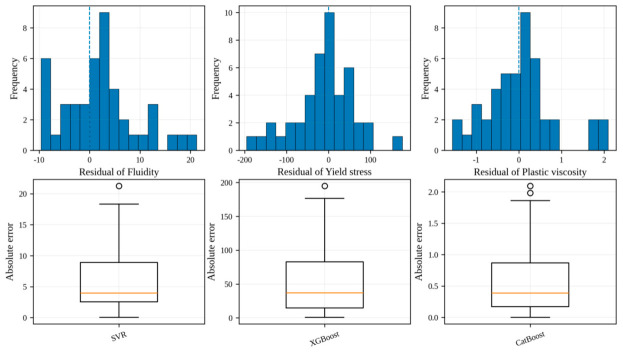
Residual distribution and error boxplots of the optimal prediction model.

The residual analysis further verifies the stability of the selected models. In Figure 17, the best fluidity model has a mean residual of 2.01 mm and an MAE of 5.84 mm, while the best yield stress model has a mean residual of −8.56 Pa and an MAE of 52.17 Pa. For viscosity, the residual distribution is the most compact, with a mean residual of only 0.018 Pa·s and an MAE of 0.595 Pa·s. The maximum absolute errors are 21.27 mm for fluidity, 195.06 Pa for yield stress, and 2.10 Pa·s for viscosity. These errors are acceptable for preliminary mix design screening, especially because rheological properties are highly sensitive to mixing sequence, temperature, shear history, and local particle packing.

### 3.4. Explainable Machine Learning Analysis and Identification of the Optimal Blending Range

**Figure 18:**
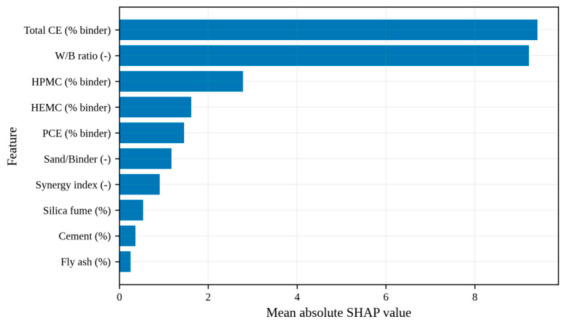
SHAP feature importance ranking for the flowability prediction model.

The SHAP analysis reveals that total cellulose ether dosage is the dominant variable for all three rheological responses. In Figure 18, the mean absolute SHAP value of total cellulose ether for fluidity is 9.414, slightly higher than that of the W/B ratio, which is 9.220. HPMC and HEMC have lower but still meaningful contributions, with SHAP values of 2.787 and 1.622, respectively. This means that the reduction in fluidity is mainly controlled by the total amount of cellulose ether, while the HPMC–HEMC ratio further adjusts the magnitude of flow loss. Similar dosage-dependent effects have been reported in recent HEMC studies on UHPC, where HEMC content and molecular chain characteristics were identified as key parameters affecting rheological behavior and hydration kinetics.

**Figure 19:**
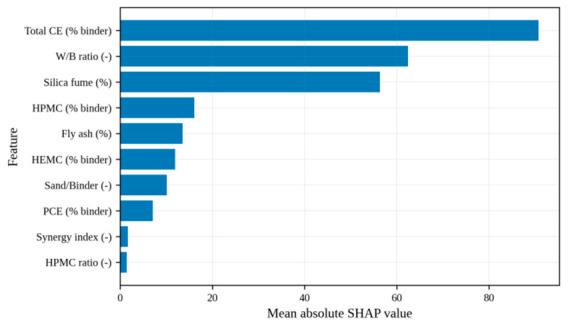
SHAP feature importance ranking for the dynamic yield stress prediction model.

**Figure 20:**
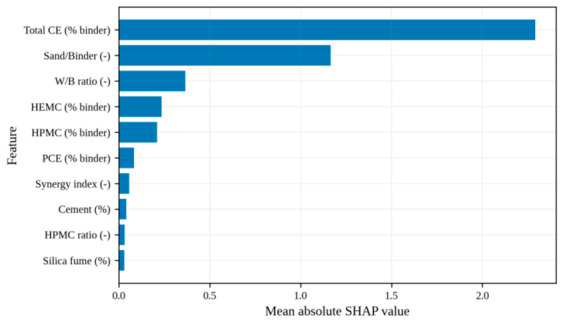
SHAP feature importance ranking for the plastic viscosity prediction model.

For dynamic yield stress, Figure 19 shows that total cellulose ether again ranks first, with a mean absolute SHAP value of 90.742 Pa. W/B ratio and silica fume follow, with values of 62.448 and 56.350 Pa, respectively. HPMC and HEMC contribute 16.093 and 11.935 Pa, suggesting that their direct effects are smaller than total dosage but still relevant for tuning the structural build-up of the paste. For plastic viscosity, Figure 20 shows a different ranking: total cellulose ether remains dominant, with a SHAP value of 2.291 Pa·s, but the sand-to-binder ratio becomes the second most important variable, with a SHAP value of 1.165 Pa·s. This indicates that viscosity is controlled not only by polymer thickening but also by solid skeleton concentration and particle interaction.

**Figure 21:**
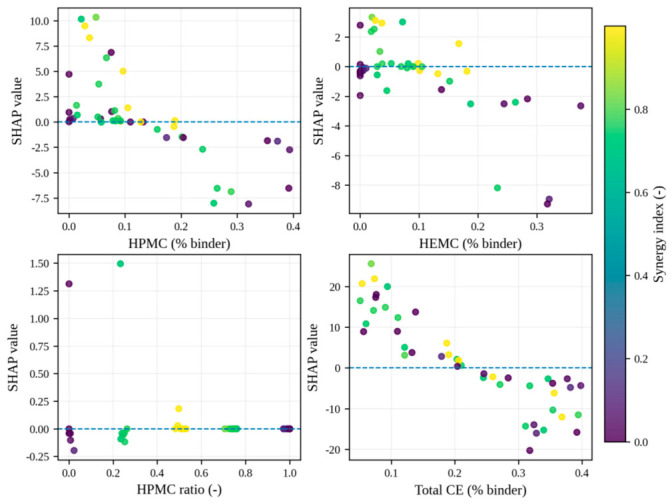
SHAP dependence plots of HPMC dosage, HEMC dosage, HPMC/HEMC ratio, and total cellulose ether dosage.

**Figure 22:**
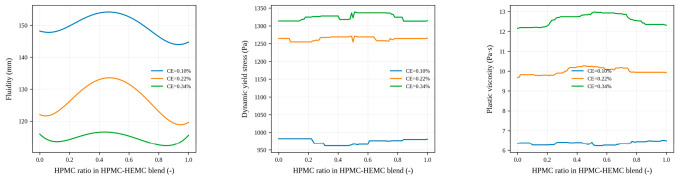
PDP curves of the effects of HPMC–HEMC blending ratio on three rheological indicators.

The dependence plots in Figure 21 provide a more detailed insight into the cellulose ether variables. Increasing total cellulose ether generally lowers fluidity and increases yield stress and viscosity, but the slope is not constant across the whole dosage range. The HPMC and HEMC dependence plots show that the strongest variation occurs at medium dosages, where the blend ratio can still modify the balance between lubrication and network formation. The PDP curves in Figure 22 further confirm the dosage effect. At a total cellulose ether dosage of 0.10%, predicted fluidity ranges from 143.97 to 154.14 mm; at 0.22%, it decreases to a range of 118.88–133.56 mm; and at 0.34%, it remains only in the range of 112.22–116.59 mm. In contrast, predicted viscosity increases from a range of about 6.23–6.50 Pa·s at 0.10% to 12.15–12.98 Pa·s at 0.34%. This behavior is consistent with recent temperature-dependent HEMC research, which showed that appropriate HEMC dosage windows are needed to maintain stable rheological performance under different environmental conditions [42].

**Figure 23:**
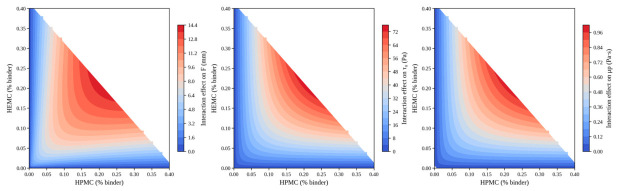
SHAP interaction effect plots between HPMC and HEMC.

The HPMC–HEMC interaction map in Figure 23 demonstrates that the interaction is strongest when both cellulose ethers are present at comparable and relatively high dosages. The maximum interaction effect appears around HPMC = 0.207% and HEMC = 0.207%, where the model predicts strong coupling among fluidity loss, yield stress increase, and viscosity enhancement. This result supports the hypothesis that HPMC–HEMC blending is not treated as a simple replacement relationship. Instead, the blend is optimized as a coupled rheology modifier, especially when the target is to simultaneously control flowability, buildability, and viscosity. Recent studies using response surface or machine learning optimization for cementitious materials have similarly emphasized that rheology-oriented mix design requires multi-parameter optimization rather than one-variable-at-a-time adjustment.

To determine the optimal HPMC–HEMC blending window, the trained ML models were used as surrogate predictors in the constrained grid-search workflow described in Section 2.5. The search was performed in the two-dimensional design space defined by total cellulose ether dosage and HPMC fraction. For each candidate point, the corresponding HPMC and HEMC dosages were calculated and then used as model inputs. The predicted fluidity, dynamic yield stress, and plastic viscosity were compared with the target ranges in Equation (24). In this study, the target ranges were set as 125–155 mm for fluidity, 980–1250 Pa for dynamic yield stress, and 7.0–11.0 Pa·s for plastic viscosity. Candidate mixtures satisfying all three conditions were defined as feasible mixtures. Among these feasible candidates, the highest overall desirability was located near a balanced HPMC–HEMC ratio of approximately 0.50, corresponding to HPMC = 0.092% and HEMC = 0.092%. Therefore, the optimal blending window was determined by the simultaneous satisfaction of the three rheological-property constraints rather than by maximizing a single output.

The obtained prediction performance was further compared with recent ML-based studies on cementitious rheology and fresh-state property prediction. Liu et al. [16] established a dataset of 233 fresh cement-based mixtures using cement, fly ash, silica fume, water, superplasticizer, HPMC, and sand as input variables, and predicted fluidity, dynamic yield stress, and plastic viscosity. Their best test R^2^ values were 0.927 for fluidity, 0.941 for dynamic yield stress, and 0.927 for plastic viscosity. In the present study, the corresponding best test R^2^ values were 0.918, 0.896, and 0.941. These values are generally comparable to those reported by Liu et al., indicating that the present dataset and models provide reliable prediction accuracy, although the current system is more complex because HPMC dosage, HEMC dosage, total cellulose ether dosage, and HPMC–HEMC blending ratio were treated as explicit descriptors. Zafar et al. [9] used 77 laboratory samples containing nano-clay, silica fume, bentonite, and methylcellulose to predict the plastic viscosity, dynamic yield stress, and static yield stress of 3D-printable cementitious materials through ensemble learning and cross-validated hyperparameter tuning. Compared with their work, the present study uses a larger dataset and focuses specifically on the dual-cellulose ether blending effect rather than general 3D-printing additives. Chen et al. [12] developed ML models using 380 recycled coarse aggregate concrete mixtures with 11 input features and reported test R^2^ values of 0.95 for dynamic yield stress and 0.93 for plastic viscosity. Although their dataset was larger, the target material system was recycled aggregate concrete rather than cellulose ether-modified composite cement paste. Therefore, the present results are consistent with the accuracy range reported in recent ML-based rheology studies, while extending the application of explainable ML to HPMC–HEMC compound modification. More importantly, unlike most previous studies that treated polymer or admixture dosage as a single input variable, this study explicitly decomposes the cellulose ether system into HPMC dosage, HEMC dosage, total cellulose ether dosage, and blending ratio, thereby enabling the identification of a physically meaningful blending window.

## 4. Conclusions

A rheology-oriented data-driven framework was developed to evaluate the synergistic influence of HPMC–HEMC blending on composite cement paste. The dataset covered 225 mixtures and included HPMC dosage, HEMC dosage, total cellulose ether dosage, HPMC ratio, W/B ratio, PCE dosage, and binder composition as input variables. The response variables showed wide ranges, with fluidity ranging from 120.0 to 214.7 mm, dynamic yield stress from 599.4 to 1350.0 Pa, and plastic viscosity from 2.00 to 16.62 Pa·s, providing a suitable basis for nonlinear modeling and explainable analysis.

The results confirmed that total cellulose ether dosage was the most influential factor controlling rheological evolution. It showed a strong negative correlation with fluidity and positive correlations with dynamic yield stress and plastic viscosity. Moderate HPMC–HEMC blending improved the balance between mobility and stability. The balanced-HEMC and balanced-HPMC groups achieved higher average fluidity, 141.89 and 141.02 mm, than the HEMC-rich group, while the balanced-HPMC group produced the highest average plastic viscosity of 9.81 Pa·s.

Different rheological targets required different optimal prediction models. SVR gave the best prediction for fluidity, where R^2^ = 0.918 and RMSE = 7.60 mm. XGBoost was most suitable for dynamic yield stress, where R^2^ = 0.896 and RMSE = 71.74 Pa. CatBoost achieved the best viscosity prediction, where R^2^ = 0.941 and RMSE = 0.816 Pa·s. These results indicate that target-specific model selection is more reliable than applying one universal model to all rheological indicators.

Explainable machine learning further revealed that total cellulose ether dosage, W/B ratio, silica fume, and sand-to-binder ratio dominated the model outputs. The strongest HPMC–HEMC interaction appeared near HPMC = 0.207% and HEMC = 0.207%. Based on the target ranges of 125–155 mm fluidity, 980–1250 Pa yield stress, and 7–11 Pa·s viscosity, 24.05% of candidate combinations satisfied all requirements. The best design was identified at HPMC = 0.092% and HEMC = 0.092%, giving a predicted fluidity value of 139.89 mm, yield stress of 1076.10 Pa, viscosity of 8.66 Pa·s, and desirability of 0.836.

## Figures and Tables

**Table 1:** Basic physical and chemical properties of raw materials.

Material	Main Index	Unit	Value
Cement	Density	g/cm^3^	2.99
	Specific surface area	m^2^/kg	317
Fly ash	${\mathrm{SiO}}_{2}$ + ${\mathrm{Al}}_{2}O_{3}$ + ${\mathrm{Fe}}_{2}O_{3}$	%	86.4
Silica fume	${\mathrm{SiO}}_{2}$ content	%	94.7
Fine aggregate	Maximum particle size	mm	2.36
PCE	Solid content	%	≥98.0
HPMC	Viscosity grade	mPa⋅s	100,000
HEMC	Viscosity grade	mPa⋅s	100,000
Water	pH	-	7.0 ± 0.5

**Table 2:** Mixture design variables and value ranges.

Variable	Symbol	Suggested Range
Water-to-binder ratio	w/b	0.28–0.42
PCE dosage	$D_{\mathrm{PCE}}$	0.05–0.50% binder
HPMC dosage	$D_{\mathrm{HPMC}}$	0–0.40% binder
HEMC dosage	$D_{\mathrm{HEMC}}$	0–0.40% binder
Total CE dosage	$D_{\mathrm{CE}}$	0.04–0.40% binder
HPMC fraction in CE	$R_{H}$	0–1
Resting time before test	$t_{r}$	5–30 min

## Data Availability

The original contributions presented in this study are included in the article. Further inquiries can be directed to the corresponding author.
